# Putting a stop to nonsense: Revisiting gene correction therapy for neurofibromatosis type 1

**DOI:** 10.1016/j.omtn.2023.08.010

**Published:** 2023-08-24

**Authors:** David H. Gutmann

**Affiliations:** 1 Department of Neurology, Washington University School of Medicine, Box 8111, 660 South Euclid Avenue, St. Louis, MO 63110, USA

Individuals with neurofibromatosis type 1 (NF1) cancer predisposition syndrome are prone to the development of benign and malignant tumors, including peripheral nerve sheath tumors (neurofibromas). Current therapy is largely directed at slowing the growth of Schwann cell-like tumors by dampening the increased mitogenic signaling that results from loss of *NF1* protein (neurofibromin) RAS pathway regulation. However, this approach often requires continued drug exposure and does not address non-RAS-pathway or non-neoplastic cell contributions to neurofibroma maintenance. As an alternative approach, Osum and colleagues leveraged a novel Ossabaw minipig model of NF1 carrying a premature termination codon (Arg1947Term) *NF1* gene mutation to explore the possibility that nonsense suppression might restore neurofibromin expression relevant to the treatment of NF1-associated tumors.^1^

In their report, Osum and colleagues provide compelling proof-of-concept demonstrations that targeting premature termination codon (PTC) mutations using readthrough compounds (e.g., gentamicin, G418) and/or nonsense-mediated decay (NMD) inhibition can restore neurofibromin expression and reduce ERK (RAS pathway) hyperactivation in *NF1*-deficient pig cutaneous neurofibroma-derived Schwann cells. Importantly, this effect was widespread, with increased neurofibromin expression detected in multiple tissues *in vivo*, including the optic nerve, potentially supporting the use of this approach for multiple NF1-associated medical problems. Additionally, they performed detailed pharmacokinetic and pharmacodynamics studies to correlate plasma levels of drug to nonsense suppression in NF1 minipig tissues. Finally, relevant to future clinical translation, they show that the combination of PTC readthrough compounds (nonsense mutation suppression therapy) and NMD silencing was the most effective strategy. Their findings raise several important issues with respect to the treatment of individuals with NF1.

First, the use of nonsense suppression and/or NMD targeting has been proposed for other genetic disorders, including Duchenne muscular dystrophy^2^ and cystic fibrosis.^3^ These approaches take advantage of the ability of numerous agents (gentamicin, G418, ataluren) to cause PTC readthrough, resulting in the production of full-length proteins or inhibit an evolutionarily conserved translation-coupled mechanism that eliminates transcripts containing PTCs (Figure 1). The latter mRNA surveillance mechanism, termed NMD, prevents the synthesis of truncated proteins with possible dominant-negative effects. There are several models for NMD that differ in their dependence on an exon junction complex (EJC) containing up-frameshift (UPF) proteins. In the situation where a messenger RNA harbors a PTC mutation, the ribosome is unable to dissociate the downstream EJCs, allowing interactions with specific NMD factors and triggering NMD.

**Figure 1:**
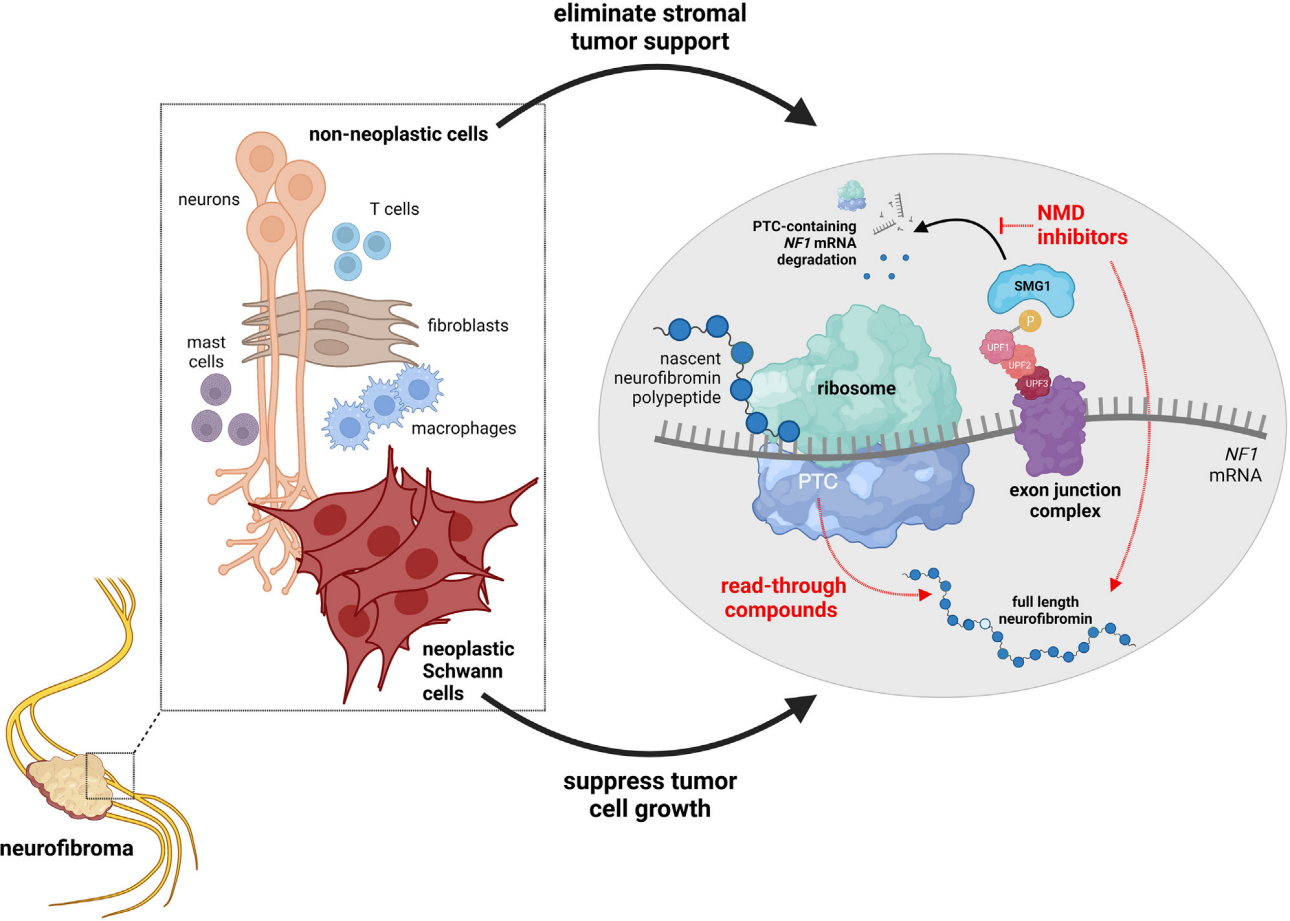
Readthrough and NMD targeting for neurofibroma therapy Benign peripheral nerve sheath tumors (plexiform and cutaneous neurofibromas) are composed of *NF1*-deficient neoplastic Schwann cell-like populations harboring biallelic *NF1* gene inactivation (no neurofibromin expression) and *NF1*-mutant non-neoplastic cell types (neurons, mast cells, macrophages, fibroblasts, and neurons) containing only one mutant *NF1* allele (reduced neurofibromin expression). During protein translation, premature termination codon (PTC) readthrough can be facilitated by compounds like G418 and gentamicin to increase neurofibromin expression. In one model of NMD, nonsense-mediated decay is initiated by the exon junction complex (EJC) containing a series of up-frameshift factors (UPF1, UPF2, UPF3), of which one (UPF1) is phosphorylated by SMG1 to ultimately culminate in the degradation of PTC-containing mRNA transcripts. NMD inhibitors block this process, resulting in increased neurofibromin expression. The effects of PTC readthrough compounds and NMD inhibitors could act on the neoplastic cell compartment to increase neurofibromin expression and reduce cell growth, as well as on the non-neoplastic cells to partly normalize neurofibromin expression and deprive the tumor cells of stromal trophic support.

Therapeutically, the combined used of NMD inhibitors and PTC readthrough compounds effectively disables the two major mechanisms that eliminate translation of *NF1*-mutant transcripts with PTCs. However, since neurofibromin forms a dimer, which is required for its function,^4^ it is not known whether there might be interfering effects of truncated or less stable neurofibromin proteins on dimer protein function. Similarly, it is not clear whether increased neurofibromin expression, potentially higher than that observed in normal cells not harboring *NF1* mutations, would have unanticipated negative effects on non-neoplastic cell function (i.e., impaired growth, differentiation, neurite extension).

Second, since 20% of individuals with NF1 harbor germline *NF1* gene PTC mutations, it is important to consider drug delivery and tissue specificity issues, specifically when entertaining treatment with aminoglycosides or NMD inhibitors. If these can be safely overcome, widespread systemic delivery, especially to the brain, could be of significant benefit to children with learning, attention, and social perception deficits, where all neurons harbor only a germline *NF1* gene mutation. Furthermore, as we learn more about the RAS-dependent^5^ and RAS-independent^6^ mechanisms that underlie NF1-associated neuronal abnormalities, normalization of neurofibromin levels in central nervous system neurons might attenuate some of the neurocognitive and behavioral symptoms common in children with NF1.

Third, while this report specifically focused on *NF1*-deficient Schwann cells, the neoplastic cells in neurofibromas, it should be appreciated that low-grade tumor growth in the setting of NF1 is heavily influenced by trophic support from non-neoplastic cells within the tumor microenvironment, including neurons and immune system cells (T lymphocytes, monocytes).^7^ Another potential use of NMD inhibition and PTC readthrough compound therapies envisions targeting the non-neoplastic cells and restoring near-normal neurofibromin levels in these *NF1*-haploinsufficient cells, resulting in loss of microenvironment (stromal) maintenance of tumor growth. In this regard, we have shown that blocking stromal trophic support in experimental murine models of low-grade optic glioma has long-lasting durable effects on tumor growth.^8^

Fourth, as we start to consider the possibility of future clinical translation for these therapies, it is important to recognize that such approaches could result in unanticipated off-target effects. For example, nonsense suppression can result in immune activation of immune cells, leading to neurobehavioral abnormalities,^9^ as well as undesired suppression of tumor suppressor gene (e.g., p53 and ATM) expression,^10^ which could unintentionally increase tumor growth and potentially induce malignant progression. Nonetheless, the proof-of-concept demonstration provided by Osum and colleagues offers new avenues for next-generation therapeutic drug design and expands our NF1 treatment toolbox.

## References

[1] Osum S.H., Oribamise E.I., Corbiere S.M.A.S., Taisto M., Jubenville T., Coutts A., Kirstein M.N., Fisher J., Moertel C., Du M. (2023). Combining nonsense mutation suppression therapy with nonsense mediated decay inhibition in Neurofibromatosis type 1. Mol. Ther. Nucleic Acids.

[2] Amar-Schwartz A., Cohen Y., Elhaj A., Ben-Hur V., Siegfried Z., Karni R., Dor T. (2023). Inhibition of nonsense-mediated mRNA decay may improve stop codon read-through therapy for Duchenne muscular dystrophy. Hum. Mol. Genet..

[3] Keating D., Marigowda G., Burr L., Daines C., Mall M.A., McKone E.F., Ramsey B.W., Rowe S.M., Sass L.A., Tullis E., VX16-445-001 Study Group (2018). VX-445-Tezacaftor-Ivacaftor in Patients with Cystic Fibrosis and One or Two Phe508del Alleles. N. Engl. J. Med..

[4] Sherekar M., Han S.W., Ghirlando R., Messing S., Drew M., Rabara D., Waybright T., Juneja P., O'Neill H., Stanley C.B. (2020). Biochemical and structural analyses reveal that the tumor suppressor neurofibromin (NF1) forms a high-affinity dimer. J. Biol. Chem..

[5] Costa R.M., Federov N.B., Kogan J.H., Murphy G.G., Stern J., Ohno M., Kucherlapati R., Jacks T., Silva A.J. (2002). Mechanism for the learning deficits in a mouse model of neurofibromatosis type 1. Nature.

[6] Anastasaki C., Wegscheid M.L., Hartigan K., Papke J.B., Kopp N.D., Chen J., Cobb O., Dougherty J.D., Gutmann D.H. (2020). Human iPSC-Derived Neurons and Cerebral Organoids Establish Differential Effects of Germline NF1 Gene Mutations. Stem Cell Rep..

[7] Guo X., Pan Y., Xiong M., Sanapala S., Anastasaki C., Cobb O., Dahiya S., Gutmann D.H. (2020). Midkine activation of CD8(+) T cells establishes a neuron-immune-cancer axis responsible for low-grade glioma growth. Nat. Commun..

[8] de Andrade Costa A., Chatterjee J., Cobb O., Cordell E., Chao A., Schaeffer S., Goldstein A., Dahiya S., Gutmann D.H. (2022). Immune deconvolution and temporal mapping identifies stromal targets and developmental intervals for abrogating murine low-grade optic glioma formation. Neurooncol. Adv..

[9] Johnson J.L., Stoica L., Liu Y., Zhu P.J., Bhattacharya A., Buffington S.A., Huq R., Eissa N.T., Larsson O., Porse B.T. (2019). Inhibition of Upf2-Dependent Nonsense-Mediated Decay Leads to Behavioral and Neurophysiological Abnormalities by Activating the Immune Response. Neuron.

[10] Supek F., Lehner B., Lindeboom R.G.H. (2021). To NMD or Not To NMD: Nonsense-Mediated mRNA Decay in Cancer and Other Genetic Diseases. Trends Genet..

